# Angioplasty in Patients with Central Venous Occlusion Prior to Device Lead Implantation

**DOI:** 10.3390/jcdd12120457

**Published:** 2025-11-25

**Authors:** Athanasios Frydas, Felix-Lucas Baehr, Henryk Dreger, Leif-Hendrik Boldt, Abdul Shokor Parwani, Gerhard Hindricks, Bernhard Gebauer, Ingo Hilgendorf, Florian Blaschke

**Affiliations:** 1 Deutsches Herzzentrum der Charité, Department of Cardiology, Angiology and Intensive Care Medicine, Augustenburger Platz 1, 13353 Berlin, Germanyhenryk.dreger@dhzc-charite.de (H.D.); ingo.hilgendorf@dhzc-charite.de (I.H.);; 2 Charité—Universitätsmedizin Berlin, Corporate Member of Freie Universität Berlin and Humboldt-Universität zu Berlin, Charitéplatz 1, 10117 Berlin, Germany; 3 DZHK (German Center for Cardiovascular Research) Partner Site Berlin, Potsdamer Str. 58, 10785 Berlin, Germany; 4 Deutsches Herzzentrum der Charité, Department of Cardiology, Angiology and Intensive Care Medicine, Charitéplatz 1, 10117 Berlin, Germany; 5 Clinic of Radiology, Charité—Universitätsmedizin Berlin, Augustenburger Platz 1, 13353 Berlin, Germany

**Keywords:** percutaneous transluminal angioplasty, cardiac implantable electronic devices, subclavian vein occlusion

## Abstract

Venous stenosis or occlusion often hinders transvenous cardiac device lead implantation, especially in patients with prior devices or long-term venous access. Surgical alternatives and femoral routes carry higher risk. We retrospectively evaluated percutaneous transluminal angioplasty (PTA) in 11 patients with significant venous obstruction. Recanalization was successful in 10/11 cases (91%). Eight leads were implanted immediately, two delayed, with one re-occlusion linked to delay. No peri-procedural complications occurred. Technical success was defined as successful lead implantation through the recanalized vein; clinical success was defined as absence of subclavian venous syndrome (no arm pain or swelling) during follow-up. During follow-up, no symptomatic re-occlusion was observed. PTA appears safe and effective, with same-session lead implantation minimizing re-occlusion risk, offering a valuable alternative when conventional venous access is not feasible.

## 1. Introduction

According to current guidelines, the left axillary or left subclavian vein are the preferred access route for transvenous cardiac device lead implantation [1,2]. If left-sided access is not feasible—due to anatomical or procedural considerations—right-sided access is an accepted alternative. The demand for cardiac implantable electronic devices (CIEDs) has steadily increased in recent years, driven by broader indications and the widespread adoption of cardiac resynchronization therapy (CRT).

However, venous access can be challenging, particularly in patients with a history of prior lead implantation or long-term central venous catheters. These interventions can lead to venous thrombosis, stenosis, or complete occlusion. Significant venous stenosis (>75%) is reported in 6–21% of patients with existing leads, while complete occlusion occurs in approximately 6–26% [3,4,5].

When venous obstruction prevents conventional transvenous lead placement, therapeutic options become limited. Surgical epicardial lead implantation via thoracotomy or sternotomy is one alternative, but it carries a higher perioperative risk and is often declined by patients. Contralateral implantation is feasible only if the contralateral venous system is patent and suitable. Implantation via the femoral vein is another option, but it is technically challenging and less commonly performed.

Percutaneous transluminal angioplasty (PTA) of an occluded subclavian vein or superior vena cava, followed by lead implantation, is a technique that offers a minimally invasive and low-risk solution in selected cases. Although some studies and recent expert consensus statements have described and recommended this approach, evidence remains limited [6,7,8,9].

Key questions remain regarding the optimal timing of angioplasty relative to lead implantation, the durability of venous patency, and the role of anticoagulation or antithrombotic therapy post-procedure.

## 2. Cases

We present 11 consecutive patients with venous occlusion or significant stenosis, who underwent percutaneous transluminal angioplasty (PTA) for transvenous lead implantation at our center between 2019 and 2025. The baseline characteristics of the patients are given in Table 1.

Seven patients required an upgrade to a cardiac resynchronization therapy (CRT) system due to heart failure with declining left ventricular ejection fraction (LVEF) caused by high ventricular pacing burden. One patient underwent reimplantation after lead extraction for device-related endocarditis, one patient underwent reimplantation after dislocation of a previously implanted lead and one patient received a first-time implantable cardioverter-defibrillator (ICD) with no prior device history.

## 3. Therapeutic Interventions

**Figure 1:**
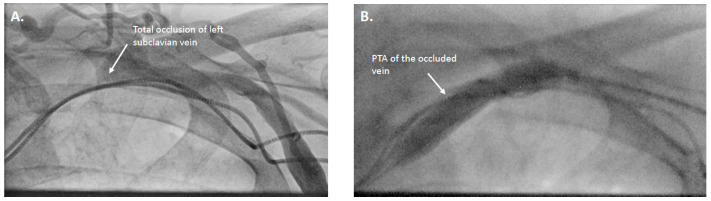
Venoplasty of a totally occluded vena subclavia. (**A**) Occlusion of the left subclavian vein as seen in venography; (**B**) Venoplasty of the occluded vein prior to device lead implantation.

**Figure 2:**
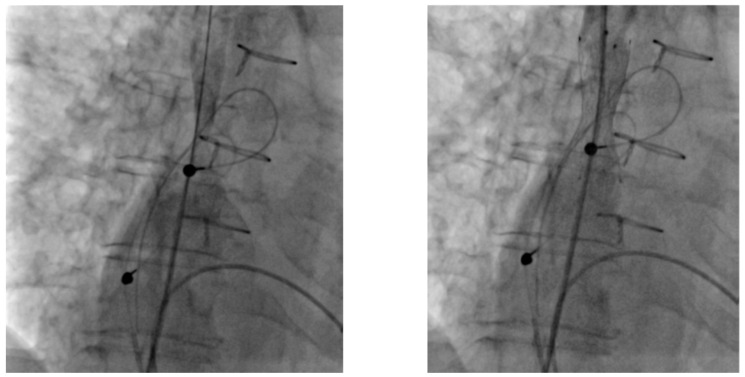
Stenting of a stenotic superior vena cava. Stenting of a severe stenosis of a superior vena cava after atrial surgery.

All PTA procedures were performed in our cardiac catheterization laboratory by an experienced interventional radiologist. Venous occlusion was defined as >75% luminal narrowing confirmed by venography. Specific catheters and wires, such as Vertebralis catheters, TIPSS needles, and Terumo stiff wires, were employed in challenging cases. Standard balloon catheters of appropriate size were used for angioplasty (5–10 mm diameter, high-pressure venous balloons), and inflation pressures ranged from 8 to 14 atm, adjusted based on lesion resistance, venographic appearance, and patient discomfort. The goal was not to fully restore the native vessel diameter, but to achieve sufficient luminal expansion to allow safe placement of a peel-away sheath for lead implantation (Figure 1); in one case, stent implantation was performed after balloon angioplasty. Subsequent device implantation was performed by an experienced interventional cardiologist. In total, 8 implantations were performed during the same session as percutaneous transluminal angioplasty (PTA), while 2 procedures were delayed by 24 h. Access strategies varied depending on anatomy and prior interventions: femoral access was used in three patients, axillary/brachial access in two, and the remainder were approached via the subclavian vein. The most commonly involved venous segments were the left subclavian and brachiocephalic veins, which were affected in seven and four patients, respectively. Superior vena cava (SVC) stenosis was identified in one patient who had previously undergone atrial surgery and required SVC stenting prior to implantation of a new lead, to ensure durable patency and prevent recurrence (Figure 2) (Table 2).

**Figure 3:**
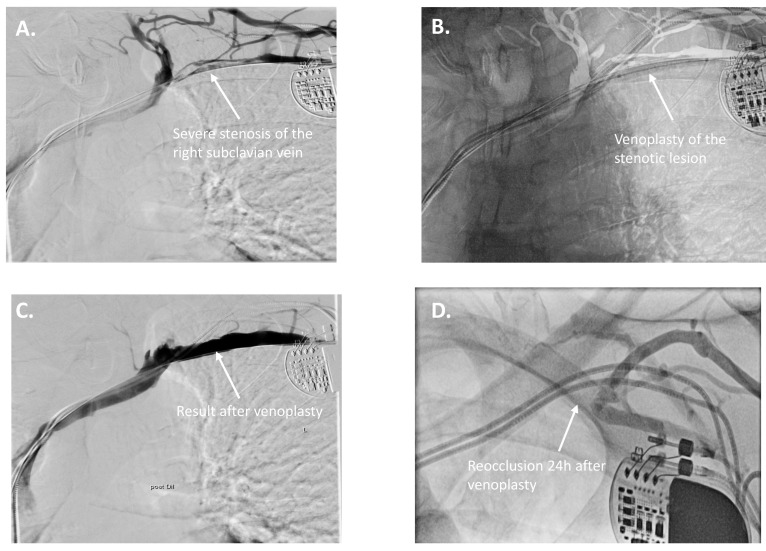
Venoplasty of a stenotic vena subclavia with re-occlusion 24 h after. (**A**) Severe stenosis of the right subclavian vein as seen in venography; (**B**) Venoplasty of the stenotic area; (**C**) Venog-raphy with excellent result post venoplasty; (**D**) Re-occlusion 24 h after the venoplasty.

Recanalization success was achieved in 10 of 11 cases, corresponding to an overall success rate of 91%. The single unsuccessful recanalization occurred due to chronic fibrotic occlusion of the left subclavian vein, despite multiple antegrade and retrograde attempts with specialized wires and catheters. In one case, although recanalization and balloon angioplasty were initially successful via axillary/brachial access, rapid re-occlusion occurred the following day (Figure 3). In one patient, although venous recanalization was successful, the coronary sinus anatomy was unsuitable for LV lead placement, and the system was upgraded to left bundle branch pacing instead (Table 2).

## 4. Follow-Up and Outcomes

No peri-procedural complications such as vascular injury or hematoma were observed. No periprocedural heparin was administered, as current evidence does not support routine heparin use during venous PTA. Eight patients were already on chronic anticoagulant therapy prior to the procedure, primarily due to atrial fibrillation or elevated thromboembolic risk. Three patients were not anticoagulated, and no anticoagulation was initiated after the procedure.

Follow-up data were available for 7 patients. No patients showed symptoms of subclavian stenosis such as pain or arm swelling. One patient experienced diaphragmatic pacing secondary to phrenic nerve stimulation, which was resolved after reprogramming (Table 3).

## 5. Discussion

Central venous obstruction remains a significant challenge in the growing population of patients requiring cardiac implantable electronic devices (CIEDs), especially with the increasing number of CRT upgrades and device revisions. In our case series, similarly to previously published studies, PTA enabled successful lead implantation in the majority of cases, supporting its utility as a practical and safe alternative to more invasive or technically demanding options such as epicardial lead placement or femoral access systems [6,7,8].

Timing of lead implantation relative to venoplasty appears to be a key determinant of procedural success. In our cohort, the majority of leads were implanted during the same session as PTA, with good outcomes. One case of re-occlusion occurred when implantation was delayed and performed via a challenging access route. This suggests that same-session implantation may reduce the risk of re-occlusion and should be considered when feasible. Stent placement was not routinely considered, except in one patient with severe SVC stenosis.

In cases involving lead extraction and re-implantation, the use of specialized extraction tools allows for the placement of a new lead through the fibrous tract created during removal. This technique can facilitate re-implantation without the need for venoplasty. In our cohort, two patients were offered this option but declined; therefore, PTA was pursued instead.

The role of anticoagulation before and after the procedure remains poorly defined and no universal recommendation exists. Most patients in our series were already anticoagulated for atrial fibrillation or increased thromboembolic risk. Previously published studies did not take a definitive stance on post-intervention anticoagulation, and re-occlusion was often considered clinically insignificant [9]. A recent study reported patients with symptomatic, lead-related venous stenosis, unresponsive to >30 days of treatment with anticoagulation, who were treated with PTA [10]. The three patients in our case series who had no other indication for anticoagulation did not receive anticoagulation after the procedure. Follow-up data revealed no clinically relevant adverse events. In general, anticoagulation strategies in venous recanalization include an initial (intra- and early post-interventional) phase, typically using unfractionated heparin (UFH) or low-molecular-weight heparin (LMWH), followed by a maintenance phase where LMWH or direct oral anticoagulants (DOAC) are now commonly preferred. A minimum of 3–6 months of therapeutic anticoagulation is generally recommended [11,12]. However, these recommendations are largely based on lower-extremity venous interventions and may not be directly applicable to upper-extremity venous recanalization with simultaneous lead implantation—a scenario where the presence of the lead itself represents a substantial risk factor for re-thrombosis [13]. In our study, one case of early re-occlusion occurred despite ongoing anticoagulation.

Compared to surgical alternatives, PTA offers a minimally invasive, lower-risk approach that preserves standard venous access and avoids thoracotomy. Contralateral implantation may not be viable in cases of bilateral obstruction or when anatomical constraints limit lead positioning. While femoral access or epicardial systems remain fallback options, they are associated with technical complexity and limited long-term data.

Our findings are consistent with previously published case series and consensus recommendations supporting the use of PTA in experienced centers. However, this study is limited by its small sample size and retrospective design. There is a clear need for larger, prospective studies to define best practices regarding procedural timing, imaging guidance, stent indications, and antithrombotic management. Standardized protocols would help streamline care and reduce variability across centers.

## 6. Conclusions

PTA is a valuable tool in the armamentarium for CIED implantation in patients with venous obstruction. Same-session device implantation appears to be safe and effective.

## Figures and Tables

**Table 1:** Baseline characteristics of the study cohort.

	Total n = 11
Age	75.4 ± 10.6
Female sex	3 (27%)
Creatinine, mg/dL	1.6 ± 1.1
Hb, mg/dL	11.8 ± 2.2
LVEF, %	34.6 ± 11.6
Hypertension	5 (45%)
Diabetes	3 (27%)
CAD	10 (91%)
Prior MI	3 (27%)
Smoker	4 (36%)
PAVK	2 (18%)
PE	0 (0%)
On anticoagulation	8 (73%)

Values are presented as mean ± standard deviation or number (percentage). CAD = coronary artery disease; MI = myocardial infarction; PAVK = peripheral arterial vascular disease; PE = pulmonary embolism.

**Table 2:** Interventions for Central Venous Occlusion or Stenosis in Patients Undergoing Device Implantation or Upgrade.

Patient	Planned Procedure	Indication	Venous Lesion	Intervention	Outcome
1	ICD → CRT-D upgrade	HFrEF, LSB, QRS 190 ms	Left subclavian vein occlusion	Attempted recanalization via right femoral vein	Recanalization unsuccessful—CRT-D not implanted
2	2-chamber pacemaker → CRT-P upgrade	HFrEF, LSB, QRS 190 ms	Left subclavian and brachiocephalic vein stenosis	Successful PTA via left subclavian vein	CRT-P successfully implanted
3	2-chamber pacemaker → CRT upgrade	HFmrEF, increased ventricular pacing	Left subclavian vein occlusion	Recanalization and PTA via axillary/brachial vein	Reocclusion next day
4	New DDD pacemaker post Candida endocarditis	Endocarditis	Superior vena cava (SVC) stenosis after atrioplasty	SVC stenting via right femoral vein, lead extraction and reimplantation	Device revision successful
5	Re-implantation of LV lead	LV lead dislocation	Venous stenosis	Successful PTA of subclavian and brachiocephalic veins via left subclavian vein	LV lead revised and repositioned
6	2-chamber pacemaker → CRT-D upgrade	LSB, HFrEF	Chronic brachiocephalic and subclavian vein occlusion	Successful recanalization and PTA	CRT-D upgrade successful
7	2-chamber pacemaker → CRT-D upgrade	LVEF deterioration, increased ventricular pacing	Right subclavian vein stenosis	Right subclavian PTA via axillary/brachial vein	CRT-D upgrade successful
8	Dual-chamber pacemaker → CRT-P upgrade	LVEF deterioration, increased ventricular pacing	Left subclavian vein occlusion	Recanalization and PTA via lateral left subclavian vein	CRT-P upgrade successful
9	2-chamber pacemaker → CRT-D upgrade	LVEF deterioration, increased ventricular pacing	Left subclavian vein occlusion	Recanalization and PTA via left subclavian vein	Recanalization successful; CRT-D upgrade not possible due to coronary sinus anatomy—upgraded to LBB stimulation system
10	Dual-chamber pacemaker → CRT-P upgrade	LSB, HFrEF	Left subclavian vein occlusion	Recanalization and PTA via left subclavian vein	CRT-P upgrade successful
11	ICD implantation	HFrEF	Right subclavian vein occlusion, left shunt arm	PTA of right brachiocephalic vein	CRT upgrade successful

Overview of 11 patients undergoing device implantation or upgrade in the setting of central venous occlusion or stenosis. The table summarizes the initial device status or planned procedure, indication for intervention, site of venous lesion, type of intervention performed, and procedural outcome. CRT = cardiac resynchronization therapy; DDD = dual-chamber pacemaker; HFrEF = heart failure with reduced ejection fraction; HFmrEF = heart failure with mildly reduced ejection fraction; ICD = implantable cardioverter defibrillator; LSB = left bundle branch block; LV = left ventricular; PTA = percutaneous transluminal angioplasty.

**Table 3:** Follow-up data of the study cohort.

Patient	Follow-Up Duration	Follow-Up Outcome
1	7 months	unremarkable findings
2	15 months	unremarkable findings
3	39 months	unremarkable findings
4	No follow-up at our center	
5	1 months	diaphragmatic stimulation, reprogramming; no further abnormalities
6	No follow-up yet	
7	No follow-up yet	
8	4 months	RV lead with complete exit block 2 months after implantation
9	No follow-up at our center	
10	4 months	LV lead impedance drop due to dislocation 2 months after implantation. Re-occlusion on the subclavian vein.
11	34 months	unremarkable findings

Follow-up data from our pacemaker outpatient clinic. The first routine follow-up is scheduled four weeks after device implantation. For patients whose implantation took place within the last four weeks, follow-up data is not yet available. The outcomes reflect the most recent findings documented in our records.

## Data Availability

The data presented in this study are not publicly available due to privacy and ethical restrictions associated with patient confidentiality and institutional regulations at Charité–Universitätsmedizin Berlin. De-identified data may be made available from the corresponding author upon reasonable request and with permission from Charité’s ethics committee.
